# Structural analysis of fungal pathogenicity-related casein kinase α subunit, Cka1, in the human fungal pathogen *Cryptococcus neoformans*

**DOI:** 10.1038/s41598-019-50678-z

**Published:** 2019-10-07

**Authors:** Belinda X. Ong, Youngki Yoo, Myeong Gil Han, Jun Bae Park, Myung Kyung Choi, Yeseul Choi, Jeon-Soo Shin, Yong-Sun Bahn, Hyun-Soo Cho

**Affiliations:** 1 Department of Systems Biology, College of Life Science and Biotechnology, Yonsei University, 50 Yonsei-ro, Seodaemun-gu, Seoul 03722 Republic of Korea; 2 Department of Biotechnology, College of Life Science and Biotechnology, Yonsei University, 50 Yonsei-ro, Seodaemun-gu, Seoul 03722 Republic of Korea; 3 Department of Microbiology, Yonsei University College of Medicine, 50-1 Yonsei-ro, Seodaemun-gu, Seoul 03722 Republic of Korea; 4 Brain Korea 21 PLUS Project for Medical Science, Yonsei University, 50-1 Yonsei-ro, Seodaemun-gu, Seoul 03722 Republic of Korea; 5 Severance Biomedical Science Institute and Institute for Immunology and Immunological Diseases, Yonsei University College of Medicine, 50-1 Yonsei-ro, Seodaemun-gu, Seoul 03722 Republic of Korea

**Keywords:** Kinases, X-ray crystallography

## Abstract

CK2α is a constitutively active and highly conserved serine/threonine protein kinase that is involved in the regulation of key cellular metabolic pathways and associated with a variety of tumours and cancers. The most well-known CK2α inhibitor is the human clinical trial candidate CX-4945, which has recently shown to exhibit not only anti-cancer, but also anti-fungal properties. This prompted us to work on the CK2α orthologue, Cka1, from the pathogenic fungus *Cryptococcus neoformans*, which causes life-threatening systemic cryptococcosis and meningoencephalitis mainly in immunocompromised individuals. At present, treatment of cryptococcosis remains a challenge due to limited anti-cryptococcal therapeutic strategies. Hence, expanding therapeutic options for the treatment of the disease is highly clinically relevant. Herein, we report the structures of Cka1-AMPPNP-Mg^2+^ (2.40 Å) and Cka1-CX-4945 (2.09 Å). Structural comparisons of Cka1-AMPPNP-Mg^2+^ with other orthologues revealed the dynamic architecture of the N-lobe across species. This may explain for the difference in binding affinities and deviations in protein-inhibitor interactions between Cka1-CX-4945 and human CK2α-CX-4945. Supporting it, *in vitro* kinase assay demonstrated that CX-4945 inhibited human CK2α much more efficiently than Cka1. Our results provide structural insights into the design of more selective inhibitors against Cka1.

## Introduction

Protein kinase CK2 (formerly known as casein kinase 2) is a constitutively active, ubiquitous and highly conserved serine/threonine kinase complex that lies within the CMGC group of protein kinases^1,2^. Given the importance of protein phosphorylation for a number of cellular processes, protein kinases are often regarded as attractive therapeutic targets and central interests of structure-based drug design^3^. Notably, CK2 is a highly pleiotropic enzyme with more than 300 substrates reported^1^. Due to its vast array of cellular targets, CK2 is involved in a diversity of complex cellular mechanisms relating to the maintenance of cell growth and cell viability^4^. More specifically, CK2 regulates several oncogenic intracellular signal transduction pathways, including the PI3K/AKT, NF-κB, and JAK-STAT pathways, which in turn alter gene expression extensively and promote cell proliferation, survival, and angiogenesis^5^.

CK2 is a heterotetrameric enzyme comprised of two catalytic subunits (CK2α) and a central dimer of regulatory subunits (CK2β)^6^. Studies have shown that the subunits consist of multiple isoforms in different organisms. In humans, three isoforms of the catalytic subunit have been discovered, namely CK2α, CK2α′ and CK2α′′, whereas only a single CK2β isoform has been identified for the regulatory subunit^7^. On the other hand, in lower eukaryotic organisms such as the budding yeast *Saccharomyces cerevisiae*, two CK2α isoforms, Cka1 and Cka2, as well as two CK2β isoforms, Ckb1 and Ckb2, have been identified^4,8^. In addition, although the CK2α subunits are regulated by the CK2β subunits, they can be catalytically active by themselves^9^. The regulatory mechanism and function of CK2 have been demonstrated by various studies^10–13^. For example, in *S*. *cerevisiae*, disruption of either *CKA1* or *CKA2* alone maintains cell viability, but disruption of both *CKA1* and *CKA2* genes causes lethality^7,10^.

Over the years, various inhibitors targeting the ATP-binding site of the CK2α subunit have been synthesised and of which, CX-4945 (5-(3-chlorophenylamino)benzo[c][2,6]naphthyridine-8-carboxylic acid; Silmitasertib) remains as the most well-known CK2α inhibitor to date^14,15^. CX-4945 is a selective inhibitor that harbours anti-tumour, anti-proliferative, and anti-angiogenic properties^14^. It functions by suppressing the pro-survival cellular PI3K/AKT pathway and stimulating apoptosis in cancer cells^14^, thereby exerting its effects on a variety of cancers and tumours such as advanced solid tumours and multiple myeloma^15^. At present, CX-4945 is the only CK2 inhibitor that has entered human clinical trials (Phase II) for cholangiocarcinoma treatment (Senhwa Biosciences, Inc.; ClinicalTrials.gov Identifier: **NCT02128282**). Hence, this indicates its clinical potential in cancer therapeutics. However, despite showing promising pharmacokinetics and pharmacodynamics profiles, there still exist limitations in regard to the use of CX-4945, such as the difficulty in achieving high selectivity and specificity. Drugs designed to inhibit CK2α activity are often associated with low selectivity due to the highly conserved ATP-binding site amongst kinases, thus, in spite of its higher selectivity than other CK2α inhibitors, CX-4945 is also relatively effective against at least twelve other kinases (nanomolar IC_50_ values) and inhibits one of them (Clk2) to a greater extent than CK2α^16^. Therefore, the need to create a more selective CK2α inhibitor cannot be undermined.

Interestingly, it was recently revealed that in addition to its anti-cancer properties, CX-4945 also exhibits anti-fungal activity against lower eukaryotes. Masłyk *et al*. demonstrated in a study that CK2α sensitises the growth of *S*. *cerevisiae* and *Candida albicans* to CX-4945, particularly inhibiting hyphal formation and cell adhesion of *C*. *albicans*^14^. Other studies have also reported on the association between cancer and fungal infections and that cancer patients undergoing intensive chemotherapy were found to be more prone to suffer from fungal diseases due to their immunosuppressed state^17,18^. Moreover, given the instability of the CK2α protein, structure-based research on CK2α is predominantly limited to *Homo sapiens* CK2α (*hs*CK2α) and *Zea mays* CK2α (*zm*CK2α), many of which are in complex with other compounds. Although the structures of *S*. *cerevisiae* Cka1 (*sc*Cka1)^8^ and *Plasmodium falciparum* CK2α (*pf*CK2α)^19^ have been recently determined, there remains a void to be filled in regard to the function and activity of CK2α in other lower eukaryotes. To bridge the gap between cancer and fungal infections, as well as to gain an understanding of the mechanisms by which fungal orthologues function as compared to their human counterpart, we have chosen to work on the *Cryptococcus neoformans* CK2α orthologue, Cka1 (*cn*Cka1), for the current study.

The basidiomycetous pathogenic fungus *C*. *neoformans* is known to be widespread in a diversity of environments, exploiting a broad range of living hosts from lower eukaryotic organisms to animals^20^. Cryptococcosis is a fungal disease caused by *C*. *neoformans*, which commonly occurs in immunocompromised individuals, and it can lead to the fatal fungal meningoencephalitis^21^. In spite of such clinical importance, treatment of cryptococcosis continues to be a challenge for researchers due to the limited availability of therapeutic strategies at present^20^. Therefore, expanding the therapeutic options for the treatment of cryptococcosis remains of high clinical relevance. In a systematic functional analysis of the kinome networks found in *C*. *neoformans*, Lee *et al*. have identified 63 pathogenicity-related kinases, some of which possess pivotal roles in cell cycle and growth control^20^. Amongst these kinases, *C*. *neoformans* possess a single CK2α orthologue, Cka1^20^, which is orthologous to both the Cka1 and Cka2 catalytic α-subunits found in *S*. *cerevisiae*^8^. Furthermore, it was reported that disruption of *CKA1* severely perturbs the production of two major virulence factors (melanin and capsule) and attenuates the pathogenicity of *C*. *neoformans*^20^. This data, along with the unveiling of CX-4945’s anti-fungal properties by Masłyk *et al*.^14^, thus prompted us to structurally analyse *C*. *neoformans* Cka1 (*cn*Cka1) to help us identify potential applications for the treatment of cancer-associated cryptococcosis.

To achieve the goal of developing anti-fungal CK2α inhibitors that target *cn*Cka1 with high selectivity, the crystal structures of *cn*Cka1-AMPPNP-Mg^2+^ (2.40 Å) and *cn*Cka1-CX-4945 (2.09 Å) were determined. Here, we demonstrate that *cn*Cka1 binds to CX-4945 at a significantly lower binding affinity than *hs*CK2α in spite of a high sequence homology. This is further substantiated on a structural basis where deviations at the ATP-binding pocket were observed. Supporting it, we found that CX-4945 inhibited *hs*CK2α much more efficiently than *cn*Cka1 (~60,000-fold difference). Our findings offer structural perspectives for the design of anti-fungal CK2α inhibitors with increased selectivity and affinity for *cn*Cka1.

## Results and Discussion

### Overall structures

**Figure 1:**
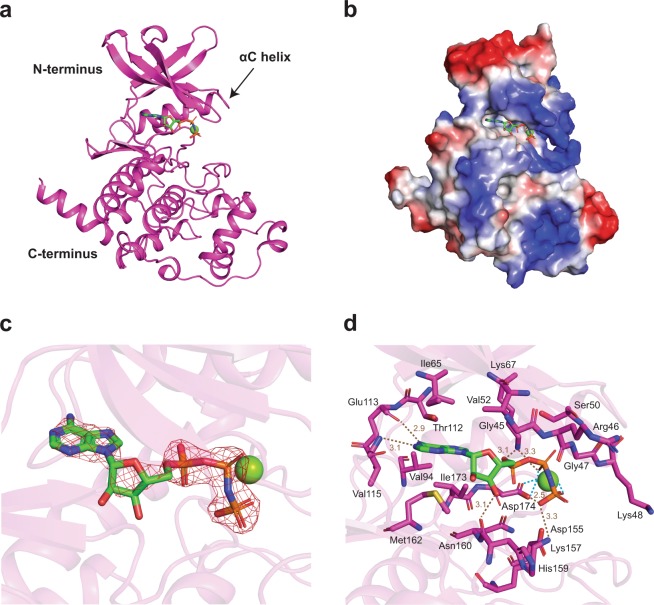
Overall structure of *cn*Cka1-AMPPNP-Mg^2+^. *cn*Cka1 is shown in magenta and AMPPNP in green. The coordinating Mg^2+^ ion is shown as a green sphere. (**a**) Structure of *cn*Cka1-AMPPNP-Mg^2+^ consisting of an ATP-binding site, N-terminal β-sheets, a critical αC helix as well as C-terminal α-helices. (**b**) Electrostatic potential map depicting the hydrophobic ATP-binding cleft where AMPPNP-Mg^2+^ lies. Basic regions are shown in blue, acidic regions in red and hydrophobic regions in white. (**c**) 2Fo-Fc map showing the binding of AMPPNP-Mg^2+^ at the ATP-binding site contoured at 2.0 σ. (**d**) Interaction between *cn*Cka1 residues and AMPPNP-Mg^2+^. Hydrogen bonds are depicted as brown dashed lines. Blue dashed lines depict the coordination of the Mg^2+^ ion by the *β*- and *γ*-phosphates of AMPPNP and Asp174.

**Figure 2:**
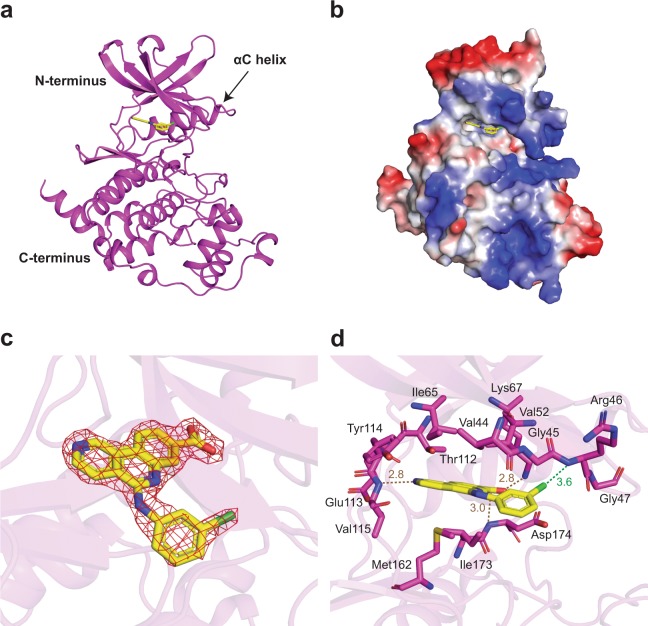
Overall structure of *cn*Cka1-CX-4945. *cn*Cka1 is shown in magenta and CX-4945 in yellow. (**a**) Structure of *cn*Cka1-CX-4945 showing its N-terminus, C-terminus and ATP-binding pocket. (**b**) Electrostatic potential map depicting the hydrophobic pocket at the ATP-binding site where the inhibitor CX-4945 binds. Basic regions are shown in blue, acidic regions in red and hydrophobic regions in white. (**c**) 2Fo-Fc map showing the binding of CX-4945 at the ATP-binding cleft contoured at 2.0 σ. (**d**) Interaction between *cn*Cka1 residues and CX-4945. Hydrogen bonds are depicted as brown dashed lines and the electrostatic interaction is depicted as a green dashed line.

We have determined the crystal structures of *cn*Cka1 in complex with AMPPNP-Mg^2+^ and CX-4945 refined to 2.40 Å and 2.09 Å, respectively (Table 1). The structures present a classic fold that is a characteristic feature of protein kinases, composing of an N-terminal region filled with several β-sheets and a critical αC helix, as well as a C-terminal region that consists primarily of α-helices (Figs 1a and 2a)^8^. The hydrophobic ATP-binding pocket lies in between these two domains. Here, the binding of the co-substrate analogue with a coordinating Mg^2+^ ion (Fig. 1b) and the inhibitor (Fig. 2b) can be observed from the electrostatic potential maps of the two structures. In addition, the 2Fo-Fc maps for these regions are also well-defined (Figs 1c and 2c).

**Table 1:** Data-collection and refinement statistics.

	*cn*Cka1-AMPPNP-Mg^2+^	*cn*Cka1-CX-4945
**Data collection statistics**		
Wavelength (Å)	0.97	0.98
Resolution range (Å)	46.67–2.4 (2.486–2.4)	47.7–2.09 (2.17–2.09)
Space group	C 2 2 2_1_	C 2 2 2_1_
**Unit cell dimensions**		
a, b, c (Å)	89.0, 95.8, 93.3	90.8, 95.4, 94.5
α, β, γ (°)	90, 90, 90	90, 90, 90
Total reflections	31893 (3138)	47336 (3733)
Unique reflections	15949 (1554)	23768 (1908)
Multiplicity	2.0 (2.0)	2.0 (2.0)
Completeness (%)	99.5 (98.9)	96.8 (78.9)
Mean I/sigma(I)	10.6 (2.35)	15.69 (2.35)
Wilson B-factor	43.2	35.02
R-merge	0.0417 (0.286)	0.0251 (0.267)
R-meas	0.0590 (0.405)	0.0355 (0.378)
R-pim	0.0417 (0.286)	0.0251 (0.267)
CC_1/2_	0.998 (0.929)	0.999 (0.884)
CC*	1 (0.981)	1 (0.969)
**Structure refinement statistics**		
Reflections used in refinement	15884 (1554)	23732 (1908)
Reflections used for R-free	792 (79)	1995 (160)
R-work	0.238 (0.381)	0.187 (0.280)
R-free	0.268 (0.406)	0.224 (0.289)
CC(work)	0.886 (0.163)	0.924 (0.521)
CC(free)	0.852 (0.376)	0.907 (0.624)
Number of non-hydrogen atoms	2812	2868
macromolecules	2745	2739
ligands	42	30
solvent	25	99
Protein residues	330	329
RMS(bonds)	0.013	0.013
RMS(angles)	1.53	1.85
Ramachandran favored (%)	97.26	97.9
Ramachandran allowed (%)	2.74	2.14
Ramachandran outliers (%)	0	0
Rotamer outliers (%)	1.68	1.35
Clashscore	7.46	3.84
Average B-factor	53.4	39.4
macromolecules	53.4	39.4
ligands	67.5	41.1
solvent	33.3	39.6

*Values in parentheses are for the highest-resolution shell.

### Structure of the *cn*Cka1-AMPPNP-Mg^2+^ complex

Full length *cn*Cka1 consists of 338 amino acids. Apart from residues 1–4 and residues 335–338, which are not visible in the electron density map, we have determined the near full-length crystal structure of *cn*Cka1 in complex with AMPPNP-Mg^2+^ (Fig. 1). The structure was elucidated using crystals grown in the presence of 10 mM AMPPNP and 10 mM MgCl_2_. The AMPPNP molecule and the coordinating Mg^2+^ ion are located in the hydrophobic ATP-binding pocket as seen from the electrostatic potential map of the structure (Fig. 1b). Figure 1d shows hydrophobic interactions between residues around the catalytic site and the AMPPNP molecule. Hydrogen bonds are also formed between the backbones of Glu113 and Val115 with the adenine group of AMPPNP; the backbone of His159 with the ribose group of AMPPNP; Lys67 with the α- and β- phosphates of AMPPNP; Asp174 and Lys157 with the γ-phosphate of AMPPNP. In addition, the Mg^2+^ ion is coordinated by the *β*- and *γ*-phosphates of AMPPNP and Asp174. In general, due to the phosphate moieties of the AMPPNP molecule, more hydrophilic interactions are observed in the AMPPNP-Mg^2+^ bound structure (Fig. 1d) as compared to the CX-4945 bound structure (Fig. 2d).

### Structure of the *cn*Cka1-CX-4945 complex

To gain an insight into the binding affinity between *cn*Cka1 and CX-4945 for the development of selective anti-fungal drugs, the near full-length structure of *cn*Cka1 in complex with CX-4945 (excluding residues 1–4 and residues 334–338 which are not visible in the electron density map) was subsequently solved and refined to 2.09 Å (Fig. 2a). Similar to the AMPPNP-Mg^2+^ bound structure, CX-4945 binds at the hydrophobic catalytic cleft (Fig. 2b) and interacts with the residues mostly through hydrophobic interactions (Fig. 2d). Additionally, hydrogen bonds are formed between Lys67 and CX-4945, as well as the backbones of Val115 and Asp174 with CX-4945, and an electrostatic interaction is formed between the backbone of Arg46 with the chloride atom of CX-4945. In general, due to the ring structures of the CX-4945 molecule, the inhibitor bound complex (Fig. 2d) contains more hydrophobic interactions as compared to the AMPPNP-Mg^2+^ bound structure (Fig. 1d).

### High similarity of the AMPPNP complex structures between species

**Figure 3:**
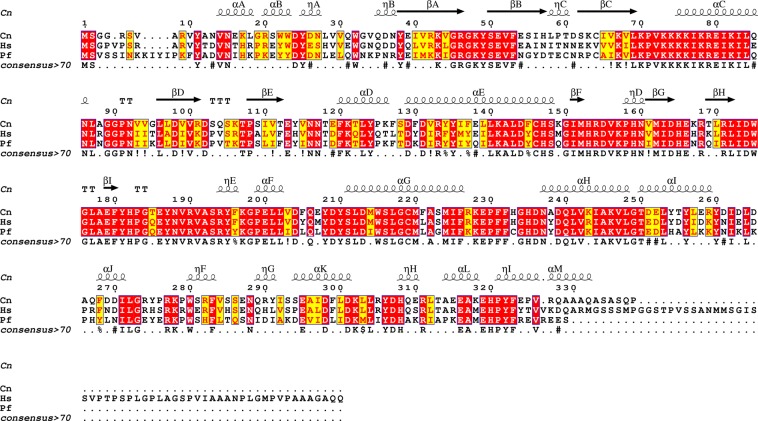
Alignment of CK2α sequences between *C*. *neoformans* (Cn), *H*. *sapie*ns (Hs) and *P*. *falciparum* (Pf) with the secondary structure of *cn*Cka1. Identical residues are shown in red and conserved residues are shown in yellow. This figure was produced using *ESPript 3*.*0* (http://espript.ibcp.fr/)^22^.

Figure 3 shows the sequence alignment between *cn*Cka1 and *hs*CK2α^22^. The sequence identity of both organisms is 70%, reflecting the high similarity between the two species. Given the high sequence homology, it is not surprising that a comparative analysis of this data along with structural analyses revealed that most of the key residues around the catalytic site are highly conserved and the overall structures of both species are almost identical with few deviations. In spite of this, in the current study, we seek to unravel differences between the human and fungal orthologues in regard to the protein-ligand interactions at the ATP-binding site. This will enable us to identify potential therapeutic strategies for the design of new inhibitors that are specific to *cn*Cka1.

**Figure 4:**
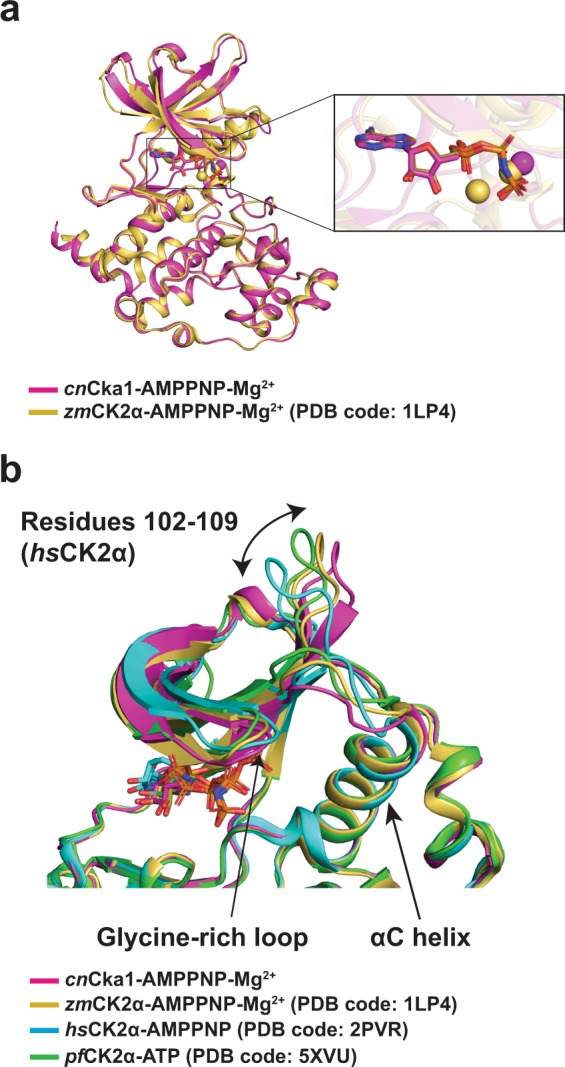
Structural comparisons among CK2α orthologues. *cn*Cka1, *zm*CK2α, *hs*CK2α and *pf*CK2α are shown in magenta, gold, cyan and green, respectively. Mg^2+^ ions are shown as spheres and the respective co-substrates (analogues) are shown as sticks in the same colour. (**a**) Superimposition of *cn*Cka1-AMPPNP-Mg^2+^ and *zm*CK2α-AMPPNP-Mg^2+^ (PDB code: **1LP4**) with a magnified view of the AMPPNP-Mg^2+^ bound catalytic site. (**b**) Structural deviations at the N-lobe of *cn*Cka1-AMPPNP-Mg^2+^, *zm*CK2α-AMPPNP-Mg^2+^ (PDB code: **1LP4**), *hs*CK2α-AMPPNP (PDB code: **2PVR**) and *pf*CK2α-ATP (PDB code: **5XVU**).

For structural analyses, we first compared the co-substrate analogue bound structures of the fungal (*cn*Cka1), maize (*zm*CK2α) and human (*hs*CK2α) orthologues. *zm*CK2α has a sequence identity of 75% with *cn*Cka1, which is slightly higher as compared to *hs*CK2α (70%). Thus, we aligned the AMPPNP-Mg^2+^ bound structures of *cn*Cka1 and *zm*CK2α (PDB code: **1LP4**) (Fig. 4a). The overall structures are highly similar and given the high sequence similarity, most of the residues at the catalytic sites were not only conserved, but also in similar positions in both structures. It is also noteworthy that the AMPPNP molecule in both structures are almost identical in orientation and position.

Superimpositions of the *cn*Cka1-AMPPNP-Mg^2+^ structure with two human complex structures, *hs*CK2α-AMPPN-Mg^2+^ (PDB code: **3NSZ**) and *hs*CK2α-AMPPNP (PDB code: **2PVR**), reveal high similarities in the overall structures too (Supplementary Fig. S1a). Similar to the maize structure, the alignment of the human structures with *cn*Cka1-AMPPNP-Mg^2+^ showed that most of the interacting residues around the catalytic cleft are conserved and located at similar positions (Supplementary Fig. S1b). For example, in the crystal structure of *cn*Cka1-AMPPNP-Mg^2+^, the two critical lysine residues, Lys67 (Lys68 in *hs*CK2α) and Lys157 (Lys158 in *hs*CK2α), which have been reported to be necessary to balance the negatively-charged moiety of the co-substrate analogue^15^, are located at the same positions as the human structure (PDB code: **2PVR**) (Supplementary Fig. S1c).

Interestingly, in the *hs*CK2α-AMPPN-Mg^2+^ structure (PDB code: **3NSZ**), Ferguson *et al*. hypothesised that the slightly acidic crystallisation environment (pH 6) resulted in the hydrolysis of AMPPNP to AMPPN and an inorganic phosphate^15^. Hence, AMPPN is observed at the catalytic site instead of AMPPNP. In contrast, the crystals of the *cn*Cka1-AMPPNP-Mg^2+^ structure were grown in a slightly alkaline environment (pH 8.5). Thus, AMPPNP hydrolysis did not occur and AMPPNP remains stably bound to the catalytic cleft (Supplementary Fig. S2a).

### Structural deviations across species when AMPPNP is bound

Surprisingly, in contrast to the almost identical orientation of the AMPPNP molecule in the *cn*Cka1-AMPPNP-Mg^2+^ and *zm*CK2α-AMPPNP-Mg^2+^ (PDB code: **1LP4**) structures (Fig. 4a), we observed that the AMPPNP molecule is in a slightly different orientation in the *hs*CK2α-AMPPNP structure (PDB code: **2PVR**) (Supplementary Fig. S2b). This is due to a difference in the position of the residues around the catalytic site, where the human structure slightly deviates from the fungal and maize structures. Therefore, this leads to a change in the interaction between the residues and the AMPPNP molecule, henceforth, causing a small deviation in the position of the AMPPNP molecule in the human complex structure.

Recently, the structure of an inactive single mutant of the *P*. *falciparum* CK2α in complex with ATP (*pf*CK2α-ATP; PDB code: **5XVU**; 64% sequence identity with *cn*Cka1; Fig. 3) was determined by Ruiz-Carrillo *et al*. where they noted several structural deviations in the N-lobe as compared to the human orthologue structure^19^. Subsequently, we superimposed the structures of *cn*Cka1-AMPPNP-Mg^2+^, *zm*CK2α-AMPPNP-Mg^2+^ (PDB code: **1LP4**) and *pf*CK2α-ATP (PDB code: **5XVU**) with *hs*CK2α-AMPPNP (PDB code: **2PVR**) and compared the region around the glycine-rich loop (Gly46-Ser51 in *hs*CK2α) where there is a shift between the βA and βB strands, the loop between βD and βE (residues 102–109 in *hs*CK2α) and the region around the αC helix (residues 70–79 in *hs*CK2α). In agreement with the paper, the N-lobe (residues 12–121 in *hs*CK2α) of the other orthologues also deviate significantly greater than the C-lobe (residues 131–329 in *hs*CK2α) in comparison with the human orthologue structure (Fig. 4b, Supplementary Table S1). The root-mean-square deviation (RMSD) values of the N-lobe are as follows: 1.77 Å (*cn*Cka1-AMPPNP-Mg^2+^), 1.88 Å (1LP4) and 2.01 Å (5XVU), while the RMSD of the C-lobe are as follows: 0.98 Å (*cn*Cka1-AMPPNP-Mg^2+^), 0.95 Å (1LP4) and 1.16 Å (5XVU). Supplementary Table S1 also shows the RMSD values for the region around the glycine-rich loop, the loop between βD and βE, and the region around the αC helix. The dynamic architecture of the N-lobe along with the difference in RMSD values across the various species suggest that different CK2α inhibitors can be designed to target individual species specifically — a property that none of the synthesised CK2α inhibitors has achieved so far, henceforth, making the current study of clinical relevance.

### The various divalent cation-binding modes of CK2α

At the catalytic site of most protein kinases, there exist two coordinating divalent cations — an essential cation, which is necessary for the catalytic activity of the kinase and interacts with the *β*- and *γ*-phosphates of the co-substrate, as well as a second cation which interacts with the *α*- and *γ*-phosphates of the co-substrate^8^. For example, two Mg^2+^ ions are visible at the ATP-binding site of the *hs*CK2α-AMPPN-Mg^2+^ structure (PDB code: **3NSZ**) when 1 mM MgCl_2_ was added^15^. In general, the essential cation is present when the concentration of the divalent cation is low and the second cation is present when the concentration of the divalent cation is high^8^. However, in the *cn*Cka1-AMPPNP-Mg^2+^ structure, despite the addition of a high concentration of MgCl_2_ (10 mM), only one Mg^2+^ ion was observed at the catalytic site to balance the charge repulsion between Asp174 and the *β*- and *γ*-phosphates of the AMPPNP molecule (Fig. 1d).

Interestingly, Liu *et al*. recently revealed that in fact, there exist multiple nucleotide-divalent cation binding modes in *S*. *cerevisiae*^8^. The authors reported that only one Mg^2+^ ion was determined in the *sc*Cka1-GMPPNP-Mg^2+^ structure (PDB code: **4JR7**) and the *sc*Cka1-ATP-Mg^2+^ structure (PDB code: **4MWH**), despite adding 0.2 M MgCl_2_ and 4 mM MgCl_2_, respectively; whereas, no Mg^2+^ ion could be identified in the *sc*Cka1-AMPPN structure (PDB code: **4JQE**) when 0.2 M MgCl_2_ was added. In an earlier study, it was also found that there were no bound divalent cations in the *hs*CK2α-AMPPNP structure (PDB code: **2PVR**) even though 2 mM MgCl_2_ was added (Supplementary Fig. S2b)^2^. Moreover, another study also indicated that no Mg^2+^ ions were visible in the AMPPNP bound *hs*CK2α subunit of the heterotetrameric *hs*CK2 structure (PDB code: **1JWH**) even though 2.0 mM MgCl_2_ was added^6^. These studies suggest that there may be a variety of divalent cation binding modes in different species of CK2α. Therefore, in future, it would be interesting to elucidate the mechanism and biochemical meaning behind the difference in interaction between Mg^2+^ ions and CK2α across species.

### CX-4945 binds to *cn*Cka1 with a lower binding affinity than *hs*CK2α despite a high sequence identity

**Figure 5:**
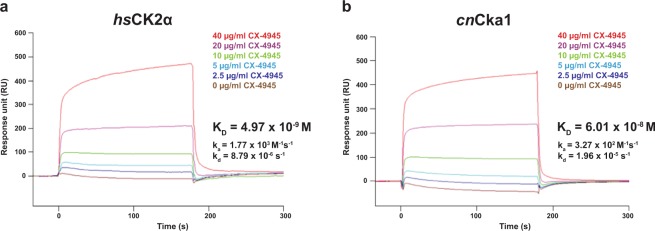
Surface plasmon resonance sensorgrams showing the interactions of *hs*CK2α and *cn*Cka1 with the inhibitor CX-4945. (**a**) Interaction between *hs*CK2α and CX-4945. (**b**) Interaction between *cn*Cka1 and CX-4945.

To explore further differences relating to the interactions at the catalytic site between the fungal and human orthologues, we next examined the binding mode between *cn*Cka1 and the inhibitor CX-4945, which has been reported to bind to *hs*CK2α with a high binding affinity (K_d_ of <10 nM)^15^. We first performed the binding assay, surface plasmon resonance (SPR), to analyse the interaction between the proteins and CX-4945, and obtained the K_D_ values of *cn*Cka1 and *hs*CK2α for CX-4945. The K_D_ value of *h*sCK2α was revealed to be 4.97 nM (Fig. 5a, Supplementary Table S2), while the K_D_ value of *cn*Cka1 was revealed to be 60.1 nM (Fig. 5b, Supplementary Table S2). Our results indicate that CX-4945 binds to *cn*Cka1 at a binding affinity about twelve times lower than *hs*CK2α. This signifies that in spite of the high sequence similarity between the fungal and human orthologues, CX-4945 binds to both orthologues at significantly different binding affinities.

### CX-4945 inhibits kinase activity of *hs*CK2α much more efficiently than *cn*Cka1

**Figure 6:**
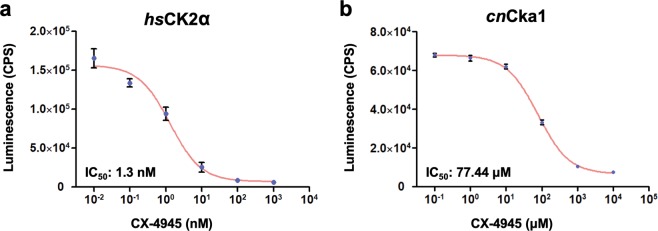
Measurement of the half maximal inhibitory concentration (IC_50_) of CX-4945 for *hs*CK2α and *cn*Cka1 through *in vitro* kinase assay. *In vitro* kinase assay of (**a**) human CK2α and (**b**) *C*. *neoformans* Cka1. The recombinant proteins were incubated with CX-4945, respectively, and consumed ATP by each kinase activity. The produced ADP was converted to ATP to be used by luciferase, and the generated luminescence was detected by a luminometer. The counts per second (CPS) is indicative of the kinase activities of *hs*CK2α and *cn*Cka1. Three-independent experiments with three-technical replicates were performed and represented in each logarithm graph. The error bar indicates ± S.E.M., and the R-square values are as follows: *hs*CK2α (0.9654); *cn*Cka1 (0.9962).

To corroborate the finding that CX-4945 binds to *hs*CK2α more efficiently than *cn*Cka1, we compared the half maximal inhibitory concentration (IC_50_) of CX-4945 for *hs*CK2α and *cn*Cka1 through *in vitro* kinase assay. In agreement with the previously reported CX-4945 IC_50_ for *hs*CK2α (~1 nM at 50 µM ATP by radiometric filter-binding assay)^23^, our *in vitro* kinase assay also showed that CX-4945 IC_50_ for *hs*CK2α at 50 µM ATP was 1.3 nM (Fig. 6a). In contrast, CX-4945 IC_50_ for *cn*Cka1 was 77.44 µM (Fig. 6b), indicating that CX-4945 IC_50_ for *hs*CK2α was ~60,000-fold lower than that for *cn*Cka1. These results indicate that CX-4945 inhibits human CK2α much more effectively than *C*. *neoformans* Cka1.

### Structural deviations in *hs*CK2α/*cn*Cka1-CX-4945 interactions

To further substantiate our findings from a structural perspective, we analysed the protein-inhibitor interactions around the ATP-binding site of the *cn*Cka1-CX-4945 and *hs*CK2α-CX-4945 structures. The structure of *hs*CK2α in complex with CX-4945 has been previously determined within the same year at 2.71 Å (PDB code: **3NGA**)^15^ and 1.60 Å (PDB code: **3PE1**)^24^. As previously mentioned, although there exists literature regarding the potency and inhibition of CX-4945 against *S*. *cerevisiae*^14^, *C*. *albicans*^14^, and *P*. *falciparum*^19^, apart from the CX-4945 bound human structures^15,24^, no other structure of the inhibitor binding to other CK2α species has been reported to date. Therefore, it is critical to investigate the binding mode of CX-4945 in lower eukaryotes on a structural basis to provide evidence for the difference in the potency of the inhibitor across species. In fact, a study has previously demonstrated that despite a sequence identity of greater than 77%, the maize and human CK2α structures differ significantly in terms of local conformations around the catalytic site when the inhibitor emodin (1,3,8-trihydroxy-6-methyl-anthraquinone) is bound, and the binding orientation of the inhibitor also changes between the two species^25^. As a consequence, we speculated that similarly, the binding mode between CX-4945 and CK2α may deviate across species.

Upon comparison of the *cn*Cka1-CX-4945 and *hs*CK2α-CX-4945 (PDB code: **3PE1**) structures, we noted some deviations around the ATP-binding site.

**Figure 7:**
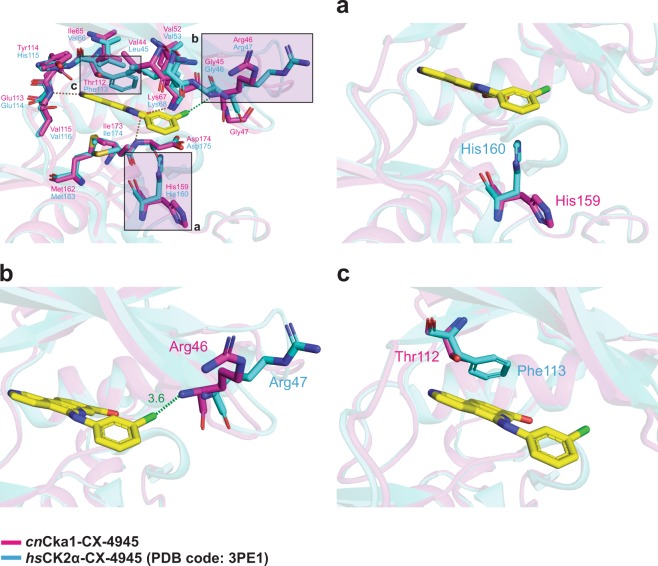
Deviations in protein-inhibitor interactions when CX-4945 is bound. The *cn*Cka1-CX-4945 structure is shown in magenta and the *hs*CK2α-CX-4945 (PDB code: **3PE1**) structure is shown in cyan. CX-4945 is shown in yellow. Hydrogen bonds are depicted as brown dashed lines and electrostatic interactions are depicted as green dashed lines. Superimposition of the *cn*Cka1-CX-4945 and *hs*CK2α-CX-4945 structures showing the residues around the catalytic cleft. (**a**) Interaction of His159 (His160 in *hs*CK2α), (**b**) Arg46 (Arg47 in *hs*CK2α) and (**c**) Thr112 (Phe113 in *hs*CK2α) with CX-4945.

In the *hs*CK2α-CX-4945 structure, a hydrophobic interaction is formed between His160 and CX-4945 (Fig. 7a). However, in the *cn*Cka1-CX-4945 structure, the corresponding residue His159 does not interact with CX-4945 as it adopts a position where it flips away from the inhibitor. Hence, His159 does not participate in any protein-inhibitor interaction unlike His160 in the human structure.

On the other hand, in the human structure, Arg47 does not contribute to any protein-inhibitor interaction, while in *cn*Cka1-CX-4945, the backbone of Arg46 interacts with the chlorine atom of the drug through an electrostatic interaction (Fig. 7b). As mentioned previously, the deviation of the N-lobe of CK2α varies across species. In fact, Arg47 is part of the glycine-rich loop of the N-lobe and is located at the tip of the βA strand — at the centre of the shift between the βA and βB strands (Fig. 4b). In the *cn*Cka1-AMPPNP-Mg^2+^ structure, we observed an RMSD value of 1.46 Å at the glycine-rich loop from the *hs*CK2α-AMPPNP structure (PDB code: **2PVR**) (Supplementary Table S1). The RMSD values of the glycine-rich loops of the *zm*CK2α-AMPPNP-Mg^2+^ (PDB code: **1LP4**) and *pf*CK2α-ATP (PDB code: **5XVU**) structures are 1.84 Å and 1.21 Å, respectively. Therefore, it can be deduced that the dynamic architecture of the N-lobe also affects to some extent the binding mode between CX-4945 and CK2α across species.

Additionally, in the *hs*CK2α-CX-4945 structure, due to the phenyl ring of Phe113, a strong hydrophobic interaction is formed between Phe113 and the inhibitor (Fig. 7c). In contrast, in the *cn*Cka1-CX-4945 structure, the hydrophobic interaction between the corresponding Thr112 residue and the inhibitor is weaker due to the lack of ring structure in the residue.

Interestingly, Thr112 (in *cn*Cka1) is also located at the “gatekeeper residue” position at the end of the βE strand^26^. Gatekeeper residues are known to play a pivotal role in the design of selective kinase inhibitors^27^ due to their positions at the ATP-binding site where they interact with residues of the R-spine^26^. While 77% of human kinases possess large gatekeeper residues (Leu, Met or Phe), only 21% possess smaller residues (Thr or Val)^26^. When the gatekeeper residue is large, accessibility of the back pocket is compromised by the bulky side chain of the residue and by the βD and βF strands, resulting in a small back pocket with restricted accessibility^27^. In contrast, the smaller side chains of small gatekeeper residues weaken the stabilisation of the R-spine^26^, and allow the expansion of the back cavity towards the βE strand and the αC helix, making the back pocket large and accessible^27^. Furthermore, because the back pocket is also a poorly conserved region at the catalytic cleft^27^, this area of plasticity thus offers opportunities for researchers to modulate the affinity and selectivity of inhibitors. While most kinase inhibitors bind directly to the ATP-binding pocket and fall under type I class of inhibitors, on the contrary, type I½ and II inhibitors are more selective as they also target the back pocket and allosteric sites, respectively, and only bind to kinases with small/medium gatekeepers^27–29^. Until now, the generation of such CK2α inhibitors was thought to be impossible due to the bulky side chain of the large gatekeeper residue (Phe113) of *hs*CK2α, which blocks the back cavity, making it inaccessible^30–32^. However, in the current study, we have observed that unlike *hs*CK2α, *cn*Cka1 has a small residue (Thr112) at the “gatekeeper residue” position. This suggests a possibility of targeting the back pocket of *cn*Cka1 for the design of selective type I½ inhibitors as the back pocket may be accessible. Since type II inhibitors also bind to kinases with small/medium gatekeepers^27^, Thr112 may therefore be exploited in future works for the development of both type I½ and II inhibitors to improve the affinity and selectivity of inhibitors for *cn*Cka1.

## Conclusion

The synthesis of highly selective CK2α inhibitors remains a challenge for scientists. With the highly conserved ATP-binding site being fully exploited for the production of the first generation of CK2α inhibitors (e.g. CX-4945), in the recent years, some groups have begun exploring other binding pockets that lie outside the ATP-binding cleft, giving rise to new generations of CK2α inhibitors^16,33–39^. As a result, the development of bivalent inhibitors as well as non-ATP competitive inhibitors has become an increasingly attractive strategy for CK2α structure-based drug design.

In a recent breakthrough, a lowly conserved novel cryptic binding site (the αD pocket) has been identified, leading to the development of second-generation inhibitors such as the bivalent inhibitor pro-CAM4066 (2016)^16^ and the αD pocket-binding inhibitor CAM4712 (2018)^33^, which displayed a more selective profile in comparison with other CK2α inhibitors. Additionally, there is an increasing number of works on the utilisation of the α/β interface for the design of new inhibitors. Some of these examples include CK2β-based small peptide inhibitors such as the conformationally constrained, disulfide-bridged 11-mer cyclic peptide Pc^34,35^; the inhibitor 5,6-dichloro-1-β-D-ribofuranosylbenzimidazole (DRB), which has a dual binding mode, allowing it to bind to both the ATP-binding site and the α/β interface^36^; the chemical inhibitor W16, which is assumed to bind to the α/β interface to inhibit the catalytic activity of CK2α through a non-ATP competitive inhibitory mechanism^37^; and more recently, two studies have reported respectively on the use of a small molecule fragment CAM187^38^ and a stable, cell-permeable peptide CAM7117^39^ to inhibit the interaction between the CK2α and CK2β subunits through the α/β interface.

However, since all these sites have been exhaustively exploited over the years, leaving little room for further improvement of current inhibitors, what lies in store for the next generation of CK2α inhibitors would be to increase their selectivity by allowing them to target specific species. To this end, our findings in this paper offer structural rationale and perspectives for the development of *cn*Cka inhibitors with greater selectivity. Both SPR and IC_50_ measurement data demonstrated that CX-4945 binds to and inhibits *hs*CK2α more efficiently than *cn*Cka1, which explains why CX-4945 only minimally inhibits the growth of *C*. *neoformans* (data not shown). However, this finding suggests a possibility that CX-4945 could be further chemically modified for optimally inhibiting *cn*Cka1 activity based on its structural information presented by this study. This potential CX-4945 derivative optimized for *cn*Cka1 could have a good anticryptococcal activity with a reasonable therapeutic index. In future, the residues that we have identified in the current study may be utilized to modify current inhibitors to increase their selectivity and affinity for *cn*Cka1. And in particular, the exploitation of Thr112 (in *cn*Cka1) may also open new opportunities for the design of selective type I½ or II inhibitors against *cn*Cka1.

## Methods

### Production of recombinant *cn*Cka1 and *hs*CK2α

The full-length DNA of *cn*Cka1 (UniProt: **J9VNH4**), which codes for amino acids Met1–Pro338, was amplified from a purified DNA clone of the cryptococcal strain *C*. *neoformans* var. grubii serotype A (H99/ATCC 208821/CBS 10515/FGSC 9487 by polymerase chain reaction (PCR) and subsequently, cloned into a pVFT3S vector (Korean patent 10-0690230) consisting of a His_6_-thioredoxin (TRX) fusion tag located at the N-terminus of the protein (pVFT3S::*cn*Cka1), using the restriction sites *BamH*I and *Xho*I, and *Escherichia coli* DH5α cells. The purified DNA clone was provided by the Seattle Structural Genomics Center for Infectious Disease (SSGCID) (Supplementary Table S3). The primers used are as follows, forward primer: CGGGATCCATGTCAGGTGGACGTAGTG and reverse primer: CCGCTCGAGTCATGGTTGAGAGGCGG. The C-terminal truncated form of human CK2α (Met1-Gly335; UniProt: **P68400**) was cloned in a similar procedure as *cn*Cka1 (pVFT3S::*hs*CK2α). The primers used are as follows, forward primer: CGGGATCCATGTCTGGTCCAGTT and reverse primer: CCGCTCGAGTTATCCCATGCGAG.

### Protein overexpression and purification

Overexpression of the *cn*Cka1 protein was induced in BL21 (DE3) competent *E*. *coli* cells by adding 0.5 mM isopropyl β-D-1-thiogalactopyranoside (IPTG) when the cell density reached an OD_600nm_ of 0.8. After incubating for 18 h at 17 °C, the cells were collected, resuspended in lysis buffer (25 mM Tris–HCl pH 7.5, 30 mM imidazole pH 7.5, 500 mM NaCl and 10% glycerol) and sonicated. Consequently, the suspension was ultracentrifuged for 1 h at 4 °C and 13,000 rpm. This was followed by incubation of the filtered supernatant in resin for 1 h on ice with gentle shaking. The resulting *cn*CKa1 protein was then purified in two steps. Ni-NTA affinity chromatography was first performed where the suspension was applied onto an Econo-Column® chromatography column (Bio-Rad). Subsequently, elution of the protein was carried out using a buffer composed of 25 mM Tris-HCl pH 7.5, 300 mM imidazole pH 7.5, 300 mM NaCl and 10% glycerol (Supplementary Fig. S3a). Subsequently, the His_6_-TRX fusion tag was removed using TEV protease at room temperature for 3.5 h (Supplementary Fig. S3b). For the next purification procedure, size exclusion chromatography using the HiLoad™ 26/600 Superdex™ 200 pg column (GE Healthcare) was performed and the bound *cn*Cka1 protein was eluted with a buffer containing 300 mM NaCl and 20 mM HEPES–NaOH pH 7.5 (Supplementary Fig. S3c). The resulting purified protein was then concentrated by ultrafiltration to 1 ml using an Amicon® Ultra-15 centrifugal filter and rebuffered to a concentration of 3 mg/ml for crystallisation trials. For surface plasmon resonance (SPR) analysis, the expression and purification procedures of the recombinant *cn*Cka1 and *hs*CK2α proteins are similar as described above. However, after the Ni-NTA affinity chromatography step, desalting and buffer exchange were carried out instead, using the HiPrep™ 26/10 Desalting column (GE Healthcare), and the proteins were eluted in the same buffer containing 300 mM NaCl and 20 mM HEPES–NaOH pH 7.5. The samples were then concentrated to about 1 mg/ml by ultrafiltration using Amicon® Ultra-15 centrifugal filters. All full-length gels are presented in Supplementary Fig. S4.

### Crystallisation

Two ligands, namely AMPPNP, which is a non-hydrolysable analogue of the co-substrate ATP of CK2α, as well as the CK2α inhibitor CX-4945 were used for crystallisation. The chemical structures of AMPPNP and CX-4945 are shown in Supplementary Figs S5a,b, respectively. *cn*Cka1-AMPPNP-Mg^2+^ and *cn*Cka1-CX-4945 were initially screened for crystallisation using the mosquito^®^ LCP crystallisation robot (TTP Labtech). The sitting drop vapor diffusion technique was subsequently carried out to obtain larger crystals and the optimised crystallisation conditions are as follows: 17 °C; 400 µl reservoir solution composed of 0.2 M lithium sulfate, 0.1 M Tris pH 8.5, 30%(*w/v*) PEG 4000; drop solution containing a mixture of 2 µl 3 mg/ml *cn*Cka1 protein, 2 µl reservoir solution and 0.5 µl Additive Screen^TM^ solution consisting of 30%(*w/v*) dextran sulfate sodium salt (Hampton Research). In addition, 10 mM MgCl_2_ and 10 mM AMPPNP were added to the drop solution to obtain *cn*Cka1-AMPPNP-Mg^2+^ crystals, while 5 mM CX-4945 was added to the drop solution to obtain *cn*Cka1-CX-4945 crystals, separately. Crystals grew to their maximum size within three weeks to a month and were cryoprotected using the reservoir solution supplemented with 20%(*v/v*) glycerol.

### Data collection, structure determination and refinement

Crystal diffraction data of *cn*Cka1-AMPPNP-Mg^2+^ were collected on beamline BL-1A of the Photon Factory (PF, KEK in Tsukuba, Japan) and diffraction data of *cn*Cka1-CX-4945 were collected on beamline 11 C of the Pohang Accelerator Laboratory (PAL in Pohang, Republic of Korea). Data sets were processed and scaled using the *HKL-2000* package^40^ and *XDS*^41^. The structures of *cn*Cka1-AMPPNP-Mg^2+^ and *cn*Cka1-CX-4945 were solved through molecular replacement using *MOLREP* of the *CCP4* program suite^42^, refined to the full resolution range via *REFMAC5*^43^ and further modelled using *Coot*^44^. The AMPPNP bound maize structure (PDB code: **1LP4**) with a sequence identity of 75% with *cn*Cka1 was used as a searched model. All structural figures were produced using *PyMOL* (The PyMOL Molecular Graphics System, Version 2.0 Schrödinger, LLC)^45^.

### Surface plasmon resonance (SPR) analysis

The *Biacore 3000* system (GE Healthcare) was used to detect the interactions of *cn*Cka1 and *hs*CK2α with CX-4945 as the analyte. His_6_-TRX_*cn*Cka1 and His_6_-TRX_*hs*CK2α proteins were immobilised onto the surface of a sensor chip CM5 (GE Healthcare). For pH scouting, a buffer composed of 10 mM sodium acetate pH 4.0 was used. The coupling process was carried out based on the molecular weight of the two proteins (MW_ligand_) and CX-4945 (MW_analyte_) to derive the R_ligand_ value. The samples were injected (10 μl/min flow rate) in a running buffer composed of 10 mM HEPES pH 7.4 and 150 mM NaCl at 17 °C and consequently replaced with the running buffer in a continuous flow rate of 10 μl/min. Sensorgrams were recorded and analysed in real time using the *Biacore 3000 Control Software* from the *Biacore 3000* system. To derive the K_D_ values, six different concentrations of CX-4945 were used for binding. All kinetic data were calculated using the *BIAevaluation Software* (GE Healthcare).

### *In vitro* kinase assay and measurement of IC_50_ of CX-4945 for *hs*CK2α and *cn*Cka1

*hs*CK2α and *cn*Cka1 kinase activities were measured using the ADP-Glo^TM^ kinase assay kit (Promega) according to the manufacturer’s instructions. The kinase reactions were performed in 25 µl volumes containing 40 mM Tris-HCl, pH 7.5, 20 mM MgCl_2_, 0.1 mg/ml BSA, 100 µM sodium vanadate, 50 µM DTT, 10 ng of each recombinant protein, 0.2 mM substrate peptide (RRRDDDSDDD, Upstate), 50 µM ATP, and 1.25 µl of various concentrations of CX-4945 dissolved in DMSO or DMSO only. CX-4945 concentrations were set between the range of 10^3^-10^−2^ nM for *hs*CK2α and 10^1^-10^−4^ mM for *cn*Cka1. The kinase reactions were incubated at room temperature for 60 mins, added with 25 µl of ADP-Glo reagent to stop the kinase reaction and remove the remaining ATP, and further incubated for 60 mins at room temperature. Subsequently, 50 µl of kinase detection reagent was added and incubated for 30 mins at room temperature. The luminescence was measured on a luminometer (VICTOR X5 Multilabel plate reader, PerkinElmer).

## Supplementary information

Supplementary information

## Data Availability

The coordinates and structure factors have been deposited in the Protein Data Bank under the accession codes 6KO6 (*cn*Cka1-AMPPNP-Mg^2+^) and 6K3L (*cn*Cka1-CX-4945). Other data are available from the corresponding author upon reasonable request.
