# Supplementary material for: Structural analysis of fungal pathogenicity-related casein kinase α subunit, Cka1, in the human fungal pathogen Cryptococcus neoformans
Source: Sci Rep. 2019 Oct 7;9:14398. doi: 10.1038/s41598-019-50678-z (PMC6779870; doi:10.1038/s41598-019-50678-z)
Supplement: Supplementary file 1 — Supplementary information [file 41598_2019_50678_MOESM1_ESM.pdf]

Supplementary information

Structural analysis of fungal pathogenicity-related casein kinase α subunit, Cka1, in the human fungal pathogen *Cryptococcus neoformans*

Belinda X. Ong, Youngki Yoo, Myeong Gil Han, Jun Bae Park, Myung Kyung Choi, Yeseul Choi, Jeon-Soo Shin, Yong-Sun Bahn, Hyun-Soo Cho

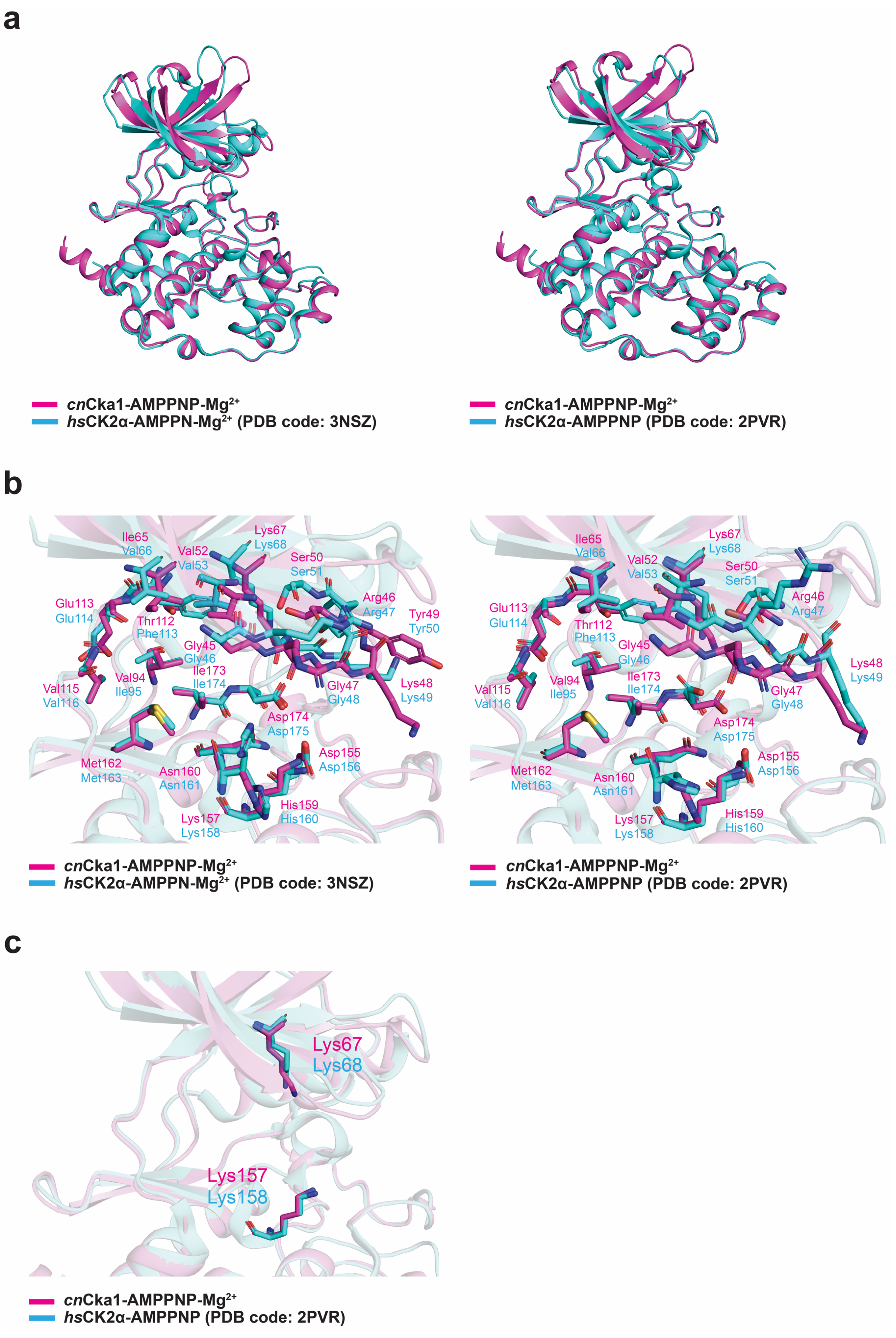
a
cnCka1-AMPPNP-Mg2+
hsCK2α-AMPPN-Mg2+ (PDB code: 3NSZ)
cnCka1-AMPPNP-Mg2+
hsCK2α-AMPPNP (PDB code: 2PVR)
b
Ile65 Val66
Val52 Val53
Lys67 Lys68
Ser50 Ser51
Glu113 Glu114
Thr112 Phe113
Arg46 Arg47
Tyr49 Tyr50
Gly45 Gly46
Ile173 Ile174
Val115 Val116
Val94 Ile95
Gly47 Gly48
Lys48 Lys49
Asp174 Asp175
Asp155 Asp156
Met162 Met163
Asn160 Asn161
His159 His160
Lys157 Lys158
cnCka1-AMPPNP-Mg2+
hsCK2α-AMPPN-Mg2+ (PDB code: 3NSZ)
cnCka1-AMPPNP-Mg2+
hsCK2α-AMPPNP (PDB code: 2PVR)
c
Lys67 Lys68
Lys157 Lys158
cnCka1-AMPPNP-Mg2+
hsCK2α-AMPPNP (PDB code: 2PVR)

Supplementary Figure S1. Structural similarities between *cn*Cka1-AMPPNP-Mg^{2+} and *hs*CK2α-AMPPN-Mg^{2+}/AMPPNP. The *cn*Cka1-AMPPNP-Mg^{2+} structure is shown in magenta and the *hs*CK2α-AMPPN-Mg^{2+}/AMPPNP structures are shown in cyan. (**a**) Superimposition of the overall structures of *cn*Cka1-AMPPNP-Mg^{2+} with *hs*CK2α-AMPPN-Mg^{2+} (PDB code: **3NSZ**) and *hs*CK2α-AMPPNP (PDB code: **2PVR**). (**b**) Residues around the active site. (**c**) Key residues Lys67 (Lys68 in *hs*CK2α) and Lys157 (Lys158 in *hs*CK2α) at the active site.

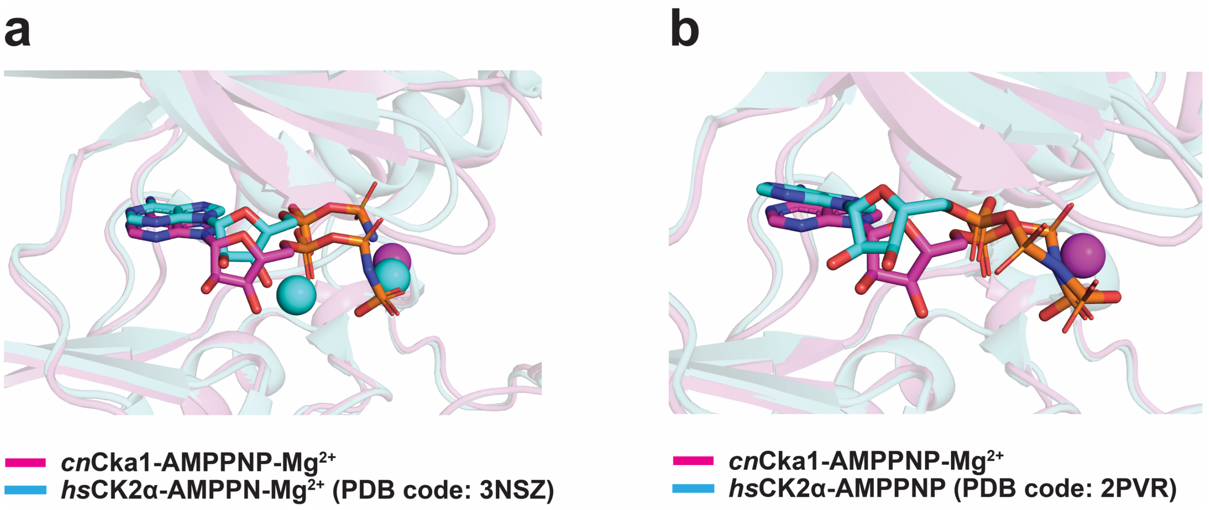

Supplementary Figure S2. The AMPPN and AMPPNP molecules at the active site. The *cn*Cka1-AMPPNP-Mg^{2+} structure is shown in magenta with AMPPNP in magenta sticks and the *hs*CK2α-AMPPN-Mg^{2+}/AMPPNP structures are shown in cyan with AMPPN and AMPPNP in cyan sticks. Mg^{2+} ions are shown as spheres. Superimposition of the active sites of *cn*Cka1-AMPPNP-Mg^{2+} with (**a**) *hs*CK2α-AMPPN-Mg^{2+} (PDB code: **3NSZ**) and (**b**) *hs*CK2α-AMPPNP (PDB code: **2PVR**).

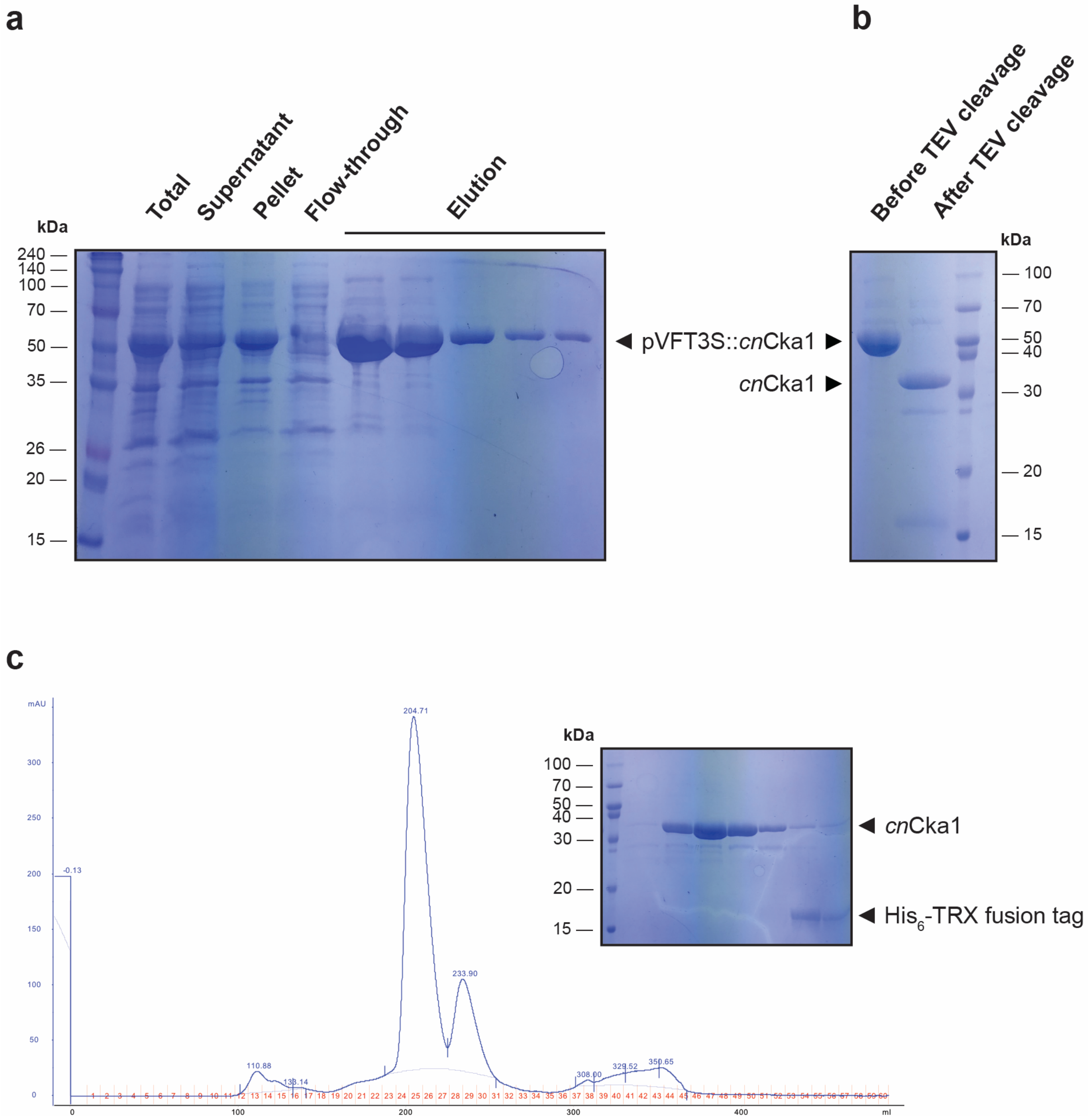

Supplementary Figure S3. Protein purification of *cn*Cka1. The sizes of pVFT3S::*cn*Cka1, *cn*Cka1 and the His_6-TRX fusion tag are 55 kDa, 40 kDa and 15 kDa, respectively. All full-length gels are presented in Supplementary Figure S4. (**a**) Ni-NTA affinity chromatography. (**b**) TEV cleavage. (**c**) Size exclusion chromatography.

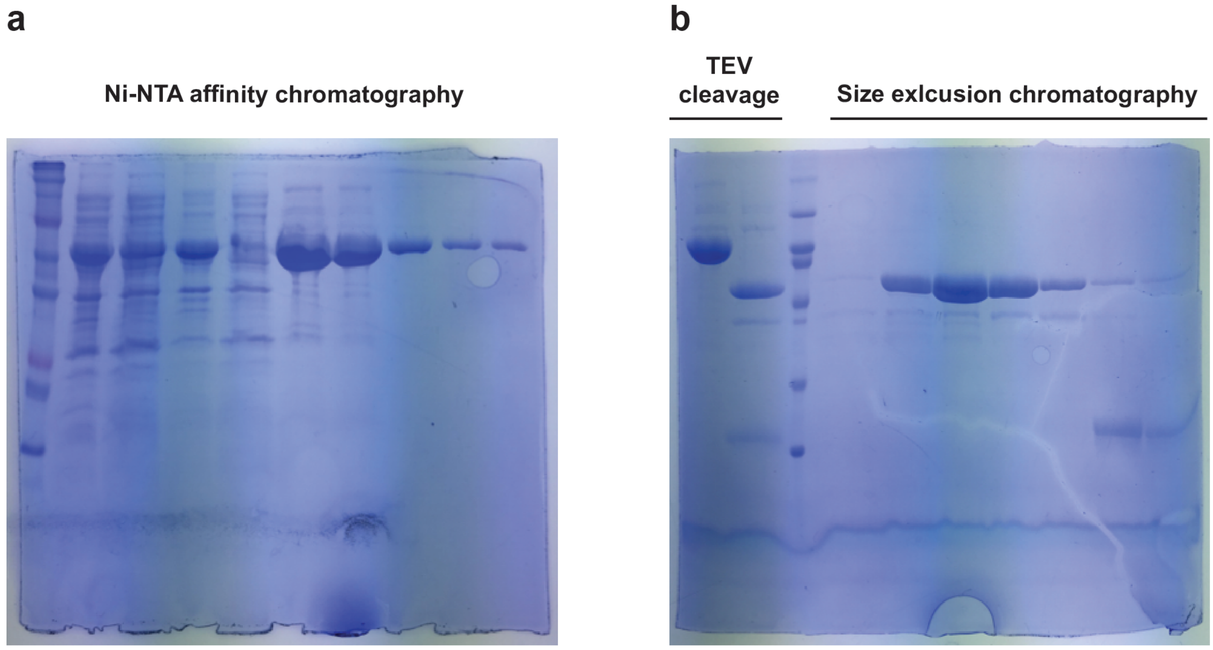

Supplementary Figure S4. Full-length view of the SDS-PAGE gels stained in Coomassie Brilliant Blue shown in Figure S3. (**a**) Ni-NTA affinity chromatography. (**b**) TEV cleavage and size exclusion chromatography.

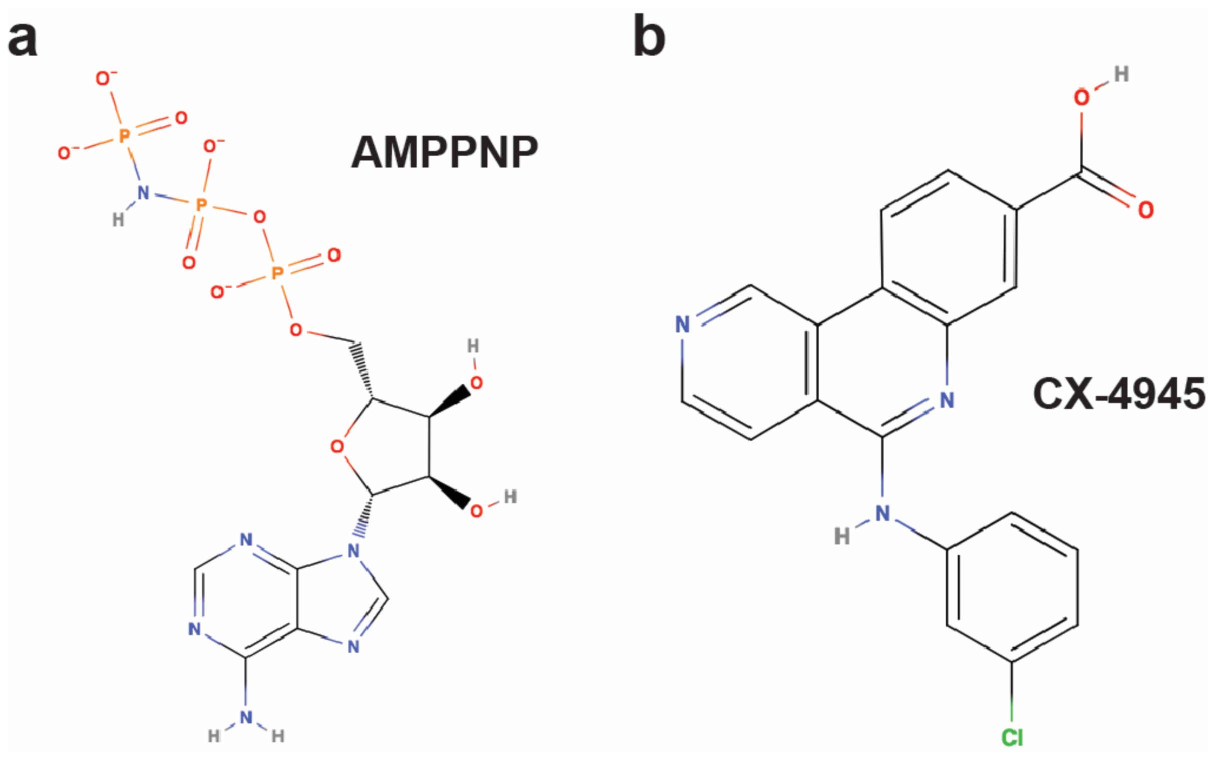

Supplementary Figure S5. Chemical structures of the co-substrate analogue and the inhibitor. (**a**) Structure of AMPPNP. (**b**) Structure of CX-4945. This figure was produced using *MolView* (molview.org/).

**Comparison with *hs*CK2α-AMPPNP (PDB code: 2PVR)**	**RMSD (Å)**		
	***cn*Cka1-AMPPNP-Mg^{2+}**	***zm*CK2α-AMPPNP-Mg^{2+} (PDB code: 1LP4)**	***pf*CK2α-ATP (PDB code: 5XVU)**
**Overall structure**	1.33	1.36	1.61
**N-lobe (residues 12-121)**	1.77	1.88	2.01
**C-lobe (residues 131-329)**	0.98	0.95	1.16
**Glycine-rich loop (Gly46-Ser51)**	1.46	1.84	1.21
**Loop between βD and βE (residues 102-109)**	1.15	1.43	1.17
**Region around the αC helix (residues 70-79)**	2.19	3.03	2.69

Supplementary Table S1. RMSD of *cn*Cka1, *zm*CK2α and *pf*CK2α when compared to *hs*CK2α.

**Protein**	**k_a ($M^{-1}s^{-1}$)**	**k_d (s^{-1})**	**K_A (M^{-1})**	**K_D (M)**
***hs*CK2α**	1.77×10^3	8.79×10^{-6}	2.01×10^8	4.97×10^{-9}
***cn*Cka1**	3.27×10^2	1.96×10^{-5}	1.66×10^7	6.01×10^{-8}

Supplementary Table S2. SPR data for the interactions of *hs*CK2α and *cn*Cka1 with the inhibitor CX-4945.

**SSGCID ID**	CrneC.00439.a.B1
**Organism**	*Cryptococcus neoformans*
**UniProt**	J9VNH4
**Gene Page**	https://apps.sbri.org/SSGCIDTargetStatus/Target/CrneC.00439.a
**Annotation**	CMGC/CK2 protein kinase

Supplementary Table S3. Information regarding the DNA clone received from SSGCID.
